# Blood Oxygen Depletion Is Independent of Dive Function in a Deep Diving Vertebrate, the Northern Elephant Seal

**DOI:** 10.1371/journal.pone.0083248

**Published:** 2013-12-23

**Authors:** Jessica U. Meir, Patrick W. Robinson, L. Ignacio Vilchis, Gerald L. Kooyman, Daniel P. Costa, Paul J. Ponganis

**Affiliations:** 1 Dept. of Anesthesia, Critical Care, and Pain Medicine, Massachusetts General Hospital/Harvard Medical School, Boston, Massachusetts, United States of America; 2 Dept. of Ecology and Evolutionary Biology, University of California Santa Cruz, Santa Cruz, California, United States of America; 3 Scripps Institution of Oceanography, University of California San Diego, La Jolla, California, United States of America; University of St Andrews, United Kingdom

## Abstract

Although energetics is fundamental to animal ecology, traditional methods of determining metabolic rate are neither direct nor instantaneous. Recently, continuous blood oxygen (O_2_) measurements were used to assess energy expenditure in diving elephant seals (*Mirounga angustirostris*), demonstrating that an exceptional hypoxemic tolerance and exquisite management of blood O_2_ stores underlie the extraordinary diving capability of this consummate diver. As the detailed relationship of energy expenditure and dive behavior remains unknown, we integrated behavior, ecology, and physiology to characterize the costs of different types of dives of elephant seals. Elephant seal dive profiles were analyzed and O_2_ utilization was classified according to dive type (overall function of dive: transit, foraging, food processing/rest). This is the first account linking behavior at this level with *in vivo* blood O_2_ measurements in an animal freely diving at sea, allowing us to assess patterns of O_2_ utilization and energy expenditure between various behaviors and activities in an animal in the wild. In routine dives of elephant seals, the blood O_2_ store was significantly depleted to a similar range irrespective of dive function, suggesting that all dive types have equal costs in terms of blood O_2_ depletion. Here, we present the first physiological evidence that all dive types have similarly high blood O_2_ demands, supporting an energy balance strategy achieved by devoting one major task to a given dive, thereby separating dive functions into distinct dive types. This strategy may optimize O_2_ store utilization and recovery, consequently maximizing time underwater and allowing these animals to take full advantage of their underwater resources. This approach may be important to optimizing energy expenditure throughout a dive bout or at-sea foraging trip and is well suited to the lifestyle of an elephant seal, which spends > 90% of its time at sea submerged making diving its most “natural” state.

## Introduction

Energy expenditure (metabolic rate) is critical to determining an animal's role within its ecosystem, from establishing a species' overall activity budget to assessing reproductive and foraging costs or physiological capabilities. Obtaining accurate measurements of energy expenditure in wild animals, however, has proven difficult. Methods ranging from doubly-labeled water techniques [1] or proxies including indices of activity level (*e.g.* accelerometry) [2] and correlations to alternative parameters (*e.g.* heart rate) [3] have been used in numerous species to assess energetic costs. None of these methods, however, provide a direct and instantaneous measure of metabolic rate. Recently, a direct and continuous method indicative of energy expenditure has been made possible, with measurements of oxygen (O_2_) depletion *via* indwelling probes in diving animals [4], [5], [6], [7]. Air-breathing, diving vertebrates ranging from penguins to sperm whales plunge to extraordinary depths of hundreds to thousands of meters, for durations of 20 to 120 minutes, enabling them to exploit unique or difficult to reach ecological niches. Despite numerous reports on the behavior and ecology of diverse divers and a growing body of physiological diving data, we still do not have a clear understanding of how diving behavior affects energy expenditure and metabolic rate in air-breathing vertebrates. A more comprehensive strategy integrating dive behavior and physiology will provide a more complete understanding of the ecology and conservation needs of these remarkable organisms.

Previous blood O_2_ measurements in diving northern elephant seals (*Mirounga angustirostris*), among the deepest and longest diving animals [8], [9], demonstrate that for this species, it is an exceptional hypoxemic tolerance and exquisite management of blood O_2_ stores that underlie their remarkable diving ability [6]. Arterial and venous O_2_ measurements, acquired with indwelling partial pressure of O_2_ (P_O2_) electrodes, revealed that in routine dives (>10 min), Pv_O2_ and Pa_O2_ reached 2–10 and 12–23 mmHg respectively [corresponding to % Hb saturation (S_O2_) of 1–26% and O_2_ contents of 0.3 (venous) and 2.7 ml O_2_ dl^−1^ blood (arterial) [6]]. These values are the lowest ever measured in a freely diving seal.

Despite such advances in the understanding of O_2_ store management while diving, detailed knowledge of specific metabolic demands at sea and activity-based O_2_ utilization strategies in diving animals remain unknown. To address this, we analyzed elephant seal dive profiles and classified O_2_ depletion data according to behavioral dive types, which have been well characterized in this species [8], [10], [11]. This is the first account linking behavior at this level with *in vivo* O_2_ measurements in an animal freely diving at sea, allowing us to assess patterns of O_2_ utilization and energy expenditure between various behaviors and activities in an animal in the wild.

Elephant seal dives have been described as fitting within 4 basic types: (*i*) v-shape (transit), (*ii*) active-bottom (pelagic foraging), (*iii*) drift (food processing or rest), and (*iv*) flat-bottom (benthic foraging or transit) dives [8], [10], [11]. The profiles and putative functions of these various dive types are different and, therefore, one might expect differences in energy expenditure. For example, locomotory costs or energy required for locating, pursuing, and capturing prey items may require greater utilization of O_2_ resources in transit and foraging dives, while food processing may be the costly activity in drift dives [12], [13], [14]. Here, we hypothesize that because elephant seals almost completely deplete their blood O_2_ stores routinely in dives greater than 10 minutes [6], the percentage of O_2_ depleted during the dive is independent of dive type. Thus, the overall activity budget may be balanced by separating activities into different dive types with similar net costs, despite differences in dive function. This may reflect a strategy to balance energy expenditure throughout the larger scale of a dive bout or at-sea foraging trip.

## Materials and Methods

Using dive analysis software (IKNOS toolbox, Y. Tremblay, unpublished), we analyzed the raw time series of depth data from MK9 time-depth recorders (TDR; Wildlife Computers, Redmond, WA; sensitive to 0.5 m, 30 g, 6.7×1.7×1.7 cm; 1 Hz sampling rate) deployed on juvenile (1- to 3-yr-old) elephant seals from the Año Nuevo colony (San Mateo County, CA) during the April molt season over three field seasons (2006, 2007, 2008) in our previous study [body mass (mean ± s.d.)  =  196±46 kg, n = 13 [6]]. All dives that were both longer than 32 s and deeper than 15 m were classified into one of four dive types (v-shape, active-bottom, drift and flat-bottom dives) following methods described previously [15]. The translocation method was used for instrument deployment [6], [16]. Briefly, this method entails removing a seal from its haul-out beach prior to the annual molt, attaching instruments, and releasing the seal at a different location, enabling tracking/measurements as the seal dives throughout its return to its haul-out to complete the molt.

### Ethics Statement

All procedures were approved by a University of California Santa Cruz Institutional Animal Care and Use Committee (IACUC) and a National Marine Fisheries Service marine mammal permit (no. 87-1743-02). All surgery was performed under general anesthesia and all efforts made to minimize discomfort to the animal.

Because the vasculature of the elephant seal is complex and circulation patterns are dynamic and still not completely understood, particularly during diving, multiple deployment sites (the aorta *via* the brachial or femoral artery, the extradural vein, and the hepatic sinus; all accessed *via* percutaneous insertion) for the P_O2_ electrode were targeted in the previous study [6]. Due to concerns of impact to the animal, only one P_O2_ electrode was deployed per seal, and each seal was subjected to a single procedure. P_O2_ profiles were obtained from 11 seals (extradural vein  =  3 seals, hepatic sinus  =  4 seals, arterial  =  4 seals) [6]. Percent blood O_2_ depletion from each site was calculated from intravascular P_O2_ electrode data from seals in the previous study [6]. This consisted of 1) Obtaining S_O2_ by applying P_O2_ data to the linear regression equation generated by the O_2_-Hb equilibrium curve (also characterized in that study) and solving for S_O2_ (at the appropriate pH; pH = 7.4 for dives < 15 min, pH = 7.3 for dives > 15 min), 2) Calculating O_2_ content using the following formula: O_2_ content (ml O_2_ dl^−1^ blood)  =  (1.34 ml O_2_ g Hb^−1^) × [Hb](g dl^−1^; measured in [6]) × S_O2_ + (0.003×P_O2_), and 3) Calculating % O_2_ depletion with the following equation: %O_2_ content depletion  =  [(maximum O_2_ content - minimum O_2_ content)/maximum O_2_ content] × 100.

Linear mixed models (REML package – R version 2.15.0 – The R Foundation for Statistical Computing) were used to assess the contributions of dive duration and dive type (fixed effects) to percent blood O_2_ depletion per dive for all dives greater than 10 minutes, with seal identity as a random factor. Analysis was limited to dives greater than 10 minutes in accordance with our hypothesis, and because such dives are considered routine for this species [9], [10], likely representing at-sea blood O_2_ depletion in free ranging elephant seals (shorter duration dives do not occur very often in migrating animals). As a significant correlation exists between % O_2_ depletion and dive duration when considering dives of all durations [6] and because some dive types were constrained to a narrow range of dive durations (active-bottom dives), we included dive duration in this analysis. To maintain physiological relevance, each site of P_O2_ electrode placement (extradural vein, hepatic sinus, or arterial) was addressed in a separate model. We used likelihood-ratio tests to compare fits of: (i) dive duration alone (ii) dive type alone, and (iii) dive duration and dive type, in order to find the most parsimonious model which accurately predicted % blood O_2_ depletion. Best-fit models were evaluated by assessing AIC and Akaike weights, defining the confidence set to include models that were within 90% of the highest Akaike weight [17], [18].

## Results

**Figure 1:**
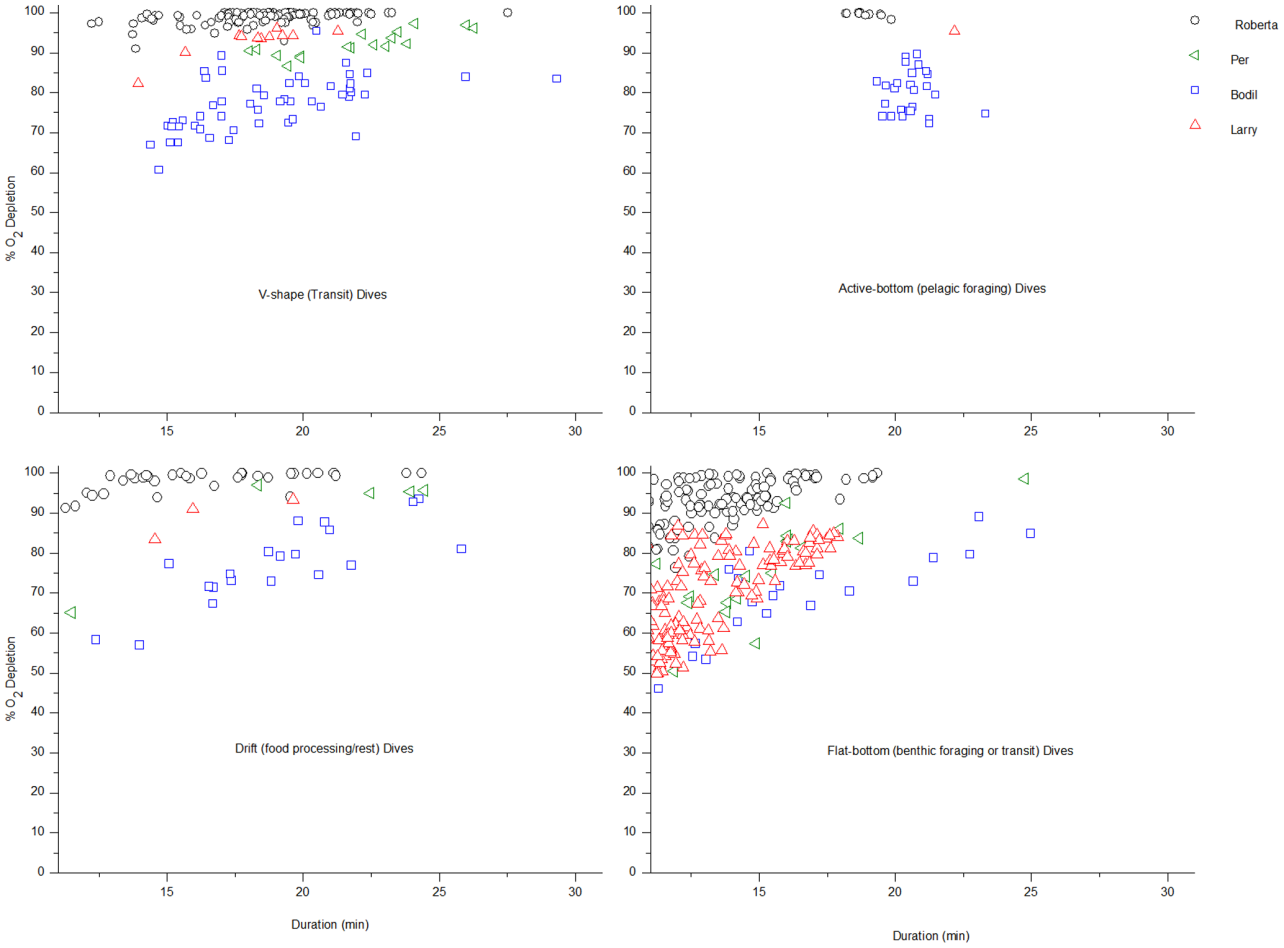
Dive duration and % blood O_2_ depletion according to dive type in diving elephant seals. Dive type panels of % blood O_2_ depletion *vs*. dive duration (min) for dives > 10 min of seals with electrodes in the hepatic sinus (n = 4 seals), symbol/color coded per individual seal. Dive types were classified according to the following: v-shape (transit), active-bottom (pelagic foraging), drift (food processing or rest), and flat-bottom (benthic foraging or transit).

**Figure 2:**
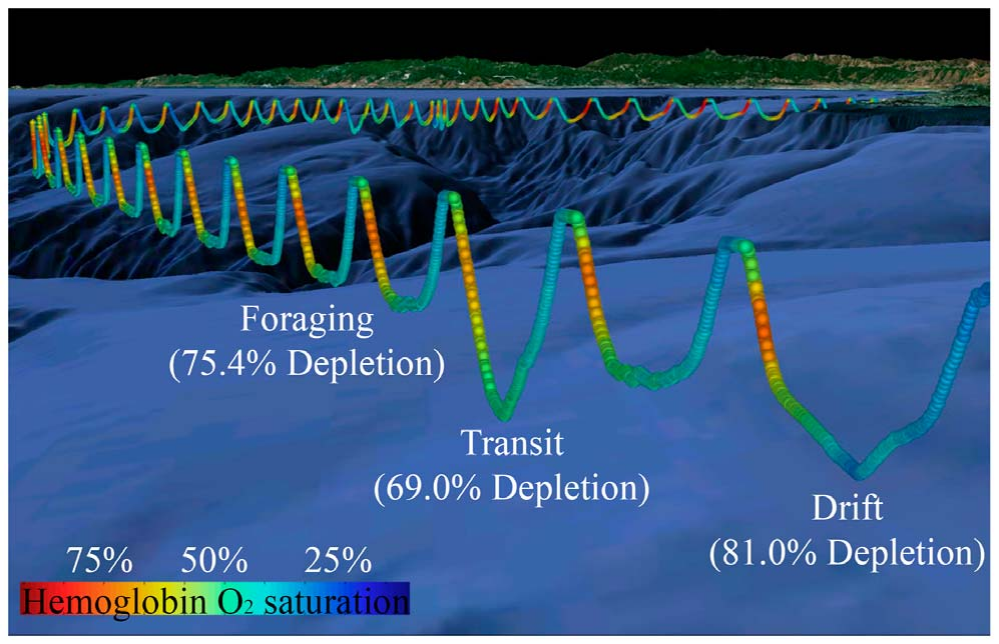
3-D dive profile with corresponding % hemoglobin saturation. A 3-D series of dives with the corresponding % hemoglobin (Hb) saturation profile during each dive from Bodil (seal with electrode in hepatic sinus) as she crosses Monterey Bay [6]. Color represents % Hb O_2_ saturation as indicated (S_O2_ decreases continuously throughout the dive, with the exception of a brief increase early in the dive. This transient increase in S_vO2_ is indicative of arterialized blood, continued gas exchange early in the dive, and the potential use of an arteriovenous shunt [6].) Note that O_2_ is depleted to a similar extent, irrespective of dive type.

The distribution of dive types was: v-shape (transit)  =  17.6%, active-bottom (pelagic foraging)  =  2.4%, drift (food processing/rest)  =  11.7%, flat-bottom (benthic foraging/transit)  =  68.3% (Table 1, Fig 1) (n = 11 seals, 1764 dives). Three seals (all with P_O2_ electrodes placed in the hepatic sinus) remained at sea for prolonged periods (9–21 days vs. 1–3.5 days for other seals), with dive profiles that included bouts of foraging and drift dives [[6], Table 1, Figs 1, 2].

**Table 1:** Breakdown of dive types for each individual seal [# dives (% of total)].

Dive Type	Starburst (EV)	Chick (EV)	Patty (EV)	Larry (HS)	Bodil (HS)	Per (HS)	Roberta (HS)	Jerry (art)	Knut (art)	Jonesie (art)	Sammy (art)	ALL SEALS
V-shape (transit)	8 (7.7)	7 (3.8)	3 (3.8)	12 (5.5)	52 (28.4)	20 (25.6)	141 (30.6)	17 (18.3)	28 (13.6)	9 (10.0)	14 (20.6)	311 (17.6)
Active-bottom (pelagic foraging)	2 (1.9)	0 (0)	0 (0)	1 (0.5)	26 (14.2)	0 (0)	10 (2.2)	1 (1.1)	1 (0.5)	1 (1.1)	0 (0)	42 (2.4)
Drift (food processing/rest)	8 (7.7)	14 (7.5)	7 (8.9)	5 (2.3)	28 (15.3)	8 (10.3)	78 (17.0)	19 (20.4)	20 (9.8)	17 (18.9)	3 (4.4)	207 (11.7)
Flat-bottom (benthic foraging/transit)	86 (82.7)	165 (88.7)	69 (87.3)	200 (91.7)	77 (42.1)	50 (64.1)	231 (50.2)	56 (60.2)	156 (76.1)	63 (70.0)	51 (75.0)	1204 (68.3)
Dive duration (min) (mean ± s.d.)	17.0±4.3	14.7±5.0	14.0±4.0	13.3±2.7	18.6±3.5	17.6±5.0	16.0±3.4	16.6±4.7	14.8±3.8	15.1±4.2	12. 4±2.4	15.6±4.1
TOTAL	104	186	79	218	183	78	460	93	205	90	68	1764

Mean dive duration is listed for dives > 10 min duration (those used for the models in this study). For O_2_ electrode deployment site, EV  =  extradural vein; HS  =  hepatic sinus; Art  =  arterial; n = 11 seals, 1764 total dives.

For each electrode site (extradural vein, hepatic sinus, arterial), the models assessing the fixed effect of dive type alone had an Akaike weight below the confidence set, resulting in exclusion as a candidate model and indicating that dive type alone cannot be considered a plausible explanation for blood O_2_ depletion. Because seals with electrodes in the hepatic sinus exhibited more diverse dive types (foraging and drift dives), detailed model results and further discussion will focus on these results (Fig 1, 2). For seals with electrodes in the hepatic sinus, the model containing both dive duration and dive type resulted in the lowest AIC value (best fit) (Table 2). According to Akaike weights, however, (all significantly < 0.90; w_i_ = 0.72, 0.28, and 2.49 * 10^−55^ for dive duration and dive type, dive duration alone, and dive type alone, respectively), none of the models can be used to make robust inferences on the dataset. As stated above, the model with dive type alone resulted in an Akaike weight so low it could not be considered a candidate model (Table 2).

**Table 2:** Model selection results.

Model	AIC	Δ_i_	Relative Likelihood [exp(−0.5*Δ_i_)]	Akaike weight (w_i_)
Dive type & Duration	4387.8	0	1	0.72
Dive Duration	4389.7	1.9	0.387	0.28
Dive Type	4638.6	250.8	3.463×10^−55^	2.5×10^−55^

The results of the model selection for data from seals with electrodes in the hepatic sinus based on Akaike's Information Criteria (AIC). Values are listed for AIC, Δ_i_ (AIC_i_ – min AIC), Relative Likelihood [exp(−0.5*Δ_i_)], and Akaike weight (w_i_; relative likelihood/Σ relative likelihood).

## Discussion

Our results demonstrate that in routine dives (> 10 min in duration) of elephant seals, the blood O_2_ store is significantly depleted to a similar range, irrespective of dive type or dive duration (Fig 1). This denotes that neither dive type nor dive duration are good predictors of blood O_2_ depletion and implies that no specific type of dive is significantly more or less costly than any other, at least in terms of blood O_2_ utilization. Such hypotheses have been proposed to account for the observation that drift dives (serving rest and food processing functions) are not significantly longer than other dives, as energy required for locomotion in other dives is thought to balance with that required for food processing in drift dives [10], [12], [14], [19]. Here, we present the first evidence that all dive types have similarly high blood O_2_ demands, supporting an energy balance strategy achieved by devoting one major task to a given dive, thereby separating dive functions into distinct dive types. Particularly since dives of elephant seals are considered to be predominantly aerobic [6], this approach may be important to optimizing energy expenditure throughout the larger scale of a dive bout or at sea foraging trip.

Although the translocation protocol typically results in short deployments consisting largely of transit dives (pelagic and benthic), some of the seals in our study (Bodil, Roberta, and Larry; all seals with electrodes in the hepatic sinus), exhibited three patterns consistent with foraging: (*i*) Longer trips. All three seals spent ≥ 9 days at sea, providing ample opportunity to locate local prey resources. (*ii*) Bouts of foraging and drift dives. We observed characteristic active-bottom dives (pelagic foraging dives) followed by bouts of drift dives, a pattern consistent with the foraging behavior observed in free-ranging juvenile and adult seals. (*iii*) Positive changes in buoyancy. Based on the previously validated method of using changes in drift rate as a measure of foraging success [15], [20], a drift dive analysis of Bodil's (the seal with the highest number of foraging dives and corresponding percentage of drift dives) dives shows at least two periods of increasing buoyancy during the 21 day deployment, consistent with an increase in adipose tissue deposition due to feeding.

Given the diverse diving behaviors exhibited by seals with electrodes in the hepatic sinus, this subset of seals provides the best example of allocating various metabolic demands into different dive types. The hepatic sinus (and posterior vena cava) is the major blood (and O_2_) reservoir in this species [21], [22], and thus likely the best index of the status of the blood O_2_ store. Regardless of regional differences in O_2_ utilization that may be associated with activities in various dive types, O_2_ depletion at this site reflects depletion from the major O_2_ reservoir. The fact that this subset of seals exhibited the widest range of dive types is perhaps fortuitous, further reinforcing our conclusions that, at least in terms of blood O_2_ utilization, all dive types have equivalent costs for elephant seals.

Although differences in O_2_ utilization at the cellular and tissue-specific levels (locomotory muscle vs. food processing organs, etc.) are certain to result from different activities, we found that the blood O_2_ budget in routine dives (> 10 minutes) is depleted to a similar range in all dive types (Figs 1,2). It is recognized that the lack of knowledge of blood flow patterns and tissue metabolic rates while diving are limiting factors in this analysis, particularly in regard to muscle blood flow and myoglobin desaturation patterns [23], [24]. For example, even given equivalent blood O_2_ depletion in different types of dives, variation in the depletion of the muscle O_2_ store during different activities would result in differences in the overall metabolic rate. Unfortunately, such measurements remain to be investigated in this consummate diver. Because blood is the dominant O_2_ reservoir (∼66%) in elephant seals [25] and dives of this species are considered predominantly aerobic in nature, we suggest that blood O_2_ saturation profiles may at least be indicative of patterns of the overall O_2_ budget, and therefore, metabolic rate. If so, all dive types may incur equal metabolic costs for elephant seals.

This proposal that different dive types may have equal metabolic costs (or at least equal blood O_2_ store depletion) is likely not applicable to all divers, particularly those with dive activities that may involve anaerobic metabolism like deep foraging sprints of pilot whales [26] or extended foraging dives in leatherback turtles [27]. Humpback whales likely incur drastically different costs for dives spent singing *vs*. energetically expensive lunge-feeding dives [28], and for Magellanic penguins, maximum depth and distance travelled are important to determining the energy expended [29]. The suggestion that the cost of a dive may be independent of dive type in elephant seals may also be at odds with the “pricing by the stroke” model proposed for the Weddell seal [30], in which the aerobic cost of the dive is correlated with locomotory costs and prey ingestion. This discrepancy, however, may reflect the very different natural histories and lifestyles of these two very capable phocid divers. The elephant seal, a predominantly aerobic diver that spends > 90% of its time at sea submerged [31], [32], is often considered a “surfacer”, while the Weddell seal is perhaps a true “diver”, often exhibiting long surface periods between dives.

For elephant seals, isolating different activities (transit, foraging, food processing, etc.) with significant O_2_ demands into distinct dive types may be a strategy to optimize O_2_ store utilization and, consequently, maximize time underwater, enabling this diver to take full advantage of its underwater resources. This approach is well suited to the lifestyle of an elephant seal. Although concepts of the separation of metabolic demands have been proposed for this species [10], [12], [14], [19], this study provides the first direct physiological evidence of such an energy balance tactic in the wild. This is also consistent with previous findings regarding activity budgets in other marine mammals. For example, grey seals delay costly physiological processes like digestion and bottlenose dolphins defer thermoregulatory costs incurred during diving to subsequent surface intervals [33], [34], [35]. These behaviors reflect the inherent conflict between the typical diving response (to conserve O_2_) and metabolic demands incurred while diving.

## References

[1] Nagy KA (2005) Field metabolic rate and body size. Journal of Experimental Biology 208: 1621–1625. 15855393 10.1242/jeb.01553

[2] Halsey LG, Shepard ELC, Wilson RP (2011) Assessing the development and application of the accelerometry technique for estimating energy expenditure. Comparative Biochemistry and Physiology a-Molecular & Integrative Physiology 158: 305–314. 10.1016/j.cbpa.2010.09.002 20837157

[3] Butler PJ (1993) To what extent can heart rate be used as an indicator of metabolic rate in free-living marine mammals? In: Boyd IL, editor. Marine Mammals: Advances in Behavioural and Population Biology. London: Zoological Society of London. pp. 317–332.

[4] Stockard TK, Heil J, Meir JU, Sato K, Ponganis KV, et al (2005) Air sac P_O2_ and oxygen depletion during dives of emperor penguins. Journal of Experimental Biology 208: 2973–2980. 16043602 10.1242/jeb.01687

[5] Ponganis PJ, Stockard TK, Meir JU, Williams CL, Ponganis KV, et al (2007) Returning on empty: extreme blood O_2_ depletion underlies dive capacity of emperor penguins. Journal of Experimental Biology 210: 4279–4285. 18055617 10.1242/jeb.011221

[6] Meir JU, Champagne CD, Costa DP, Williams CL, Ponganis PJ (2009) Extreme hypoxemic tolerance and blood oxygen depletion in diving elephant seals. American Journal of Physiology - Regulatory, Integrative, and Comparative Physiology 297: R927–R939. 10.1152/ajpregu.00247.2009 19641132

[7] Williams CL, Meir JU, Ponganis PJ (2011) What triggers the aerobic dive limit? Patterns of muscle oxygen depletion during dives of emperor penguins. Journal of Experimental Biology 214: 1802–1812. 21562166 10.1242/jeb.052233 PMC3092726

[8] Le Boeuf BJ, Costa DP, Huntley AC, Feldkamp SD (1988) Continuous, deep diving in female northern elephant seals, *Mirounga angustirostris* . Canadian Journal of Zoology 66: 446–458.

[9] Le Boeuf BJ, Morris PA, Blackwell SA, Crocker DE, Costa DP (1996) Diving behavior of juvenile northern elephant seals. Canadian Journal of Zoology 74: 1632–1644.

[10] Le Boeuf BJ, Naito Y, Asaga T, Crocker D, Costa DP (1992) Swim Speed in a Female Northern Elephant Seal Metabolic and Foraging Implications. Canadian Journal of Zoology 70: 786–794.

[11] Robinson PW, Costa DP, Crocker DE, Gallo-Reynoso JP, Champagne CD, et al.. (2012) Foraging Behavior and Success of a Mesopelagic Predator in the Northeast Pacific Ocean: Insights from a Data-Rich Species, the Northern Elephant Seal. Plos One 7. 10.1371/journal.pone.0036728 PMC3352920 22615801

[12] Crocker DE, Le Boeuf BJ, Costa DP (1997) Drift diving in female northern elephant seals: Implications for food processing. Canadian Journal of Zoology 75: 27–39.

[13] Mitani Y, Andrews RD, Sato K, Kato A, Naito Y, et al (2010) Three-dimensional resting behaviour of northern elephant seals: drifting like a falling leaf. Biology Letters 6: 163–166. 19864274 10.1098/rsbl.2009.0719 PMC2865059

[14] Le Boeuf BJ (1994) Variation in the diving pattern of northern elephant seals with age, mass, sex, and reproductive condition. Elephant seals: Population ecology, behavior, and physiology. pp. 237–252.

[15] Robinson PW, Simmons SE, Crocker DE, Costa DP (2010) Measurements of foraging success in a highly pelagic marine predator, the northern elephant seal. Journal of Animal Ecology 79: 1146–1156. 20673236 10.1111/j.1365-2656.2010.01735.x

[16] Oliver GW, Morris PA, Thorson PH, Le Boeuf BJ (1998) Homing behavior of juvenile northern elephant seals. Marine Mammal Science 14: 245–256.

[17] Royall RM (1997) Statistical evidence: a likelihood paradigm. New York: Chapman and Hall.

[18] Burnham KP, Anderson D.R. (2002) Model selection and inference: a practical information-theoretic approach, second edition. New York: Springer-Verlag.

[19] Crocker DE, Le Boeuf BJ, Naito Y, Asaga T, Costa DP (1994) Swim speed and dive function in a female northern elephant seal. Elephant seals: Population ecology, behavior, and physiology. pp. 328–339.

[20] Biuw M, McConnell B, Bradshaw CJA, Burton H, Fedak M (2003) Blubber and buoyancy: monitoring the body condition of free-ranging seals using simple dive characteristics. Journal of Experimental Biology 206: 3405–3423. 12939372 10.1242/jeb.00583

[21] Elsner RW, Scholander PF, Craig AB, Dimond EG, Irving L, et al (1964) A venous blood oxygen reservoir in the diving elephant seal. The Physiologist 7: 124.

[22] Elsner RW, Gooden B (1983) Diving and Asphyxia: a comparative study of animals and man. Cambridge: Cambridge University Press. 175 p. 6685226

[23] Davis RW, Kanatous SB (1999) Convective oxygen transport and tissue oxygen consumption in Weddell seals during aerobic dives. Journal of Experimental Biology 202: 1091–1113. 10101108 10.1242/jeb.202.9.1091

[24] Ponganis PJ, Meir JU, Williams CL (2011) In pursuit of Irving and Scholander: a review of oxygen store management in seals and penguins. Journal of Experimental Biology 214: 3325–3339. 21957096 10.1242/jeb.031252

[25] Ponganis PJ, Kooyman GL, Ridgway SH (2003) Comparative Diving Physiology. In: Brubakk AO, Neuman TS, editors. Physiology and Medicine of Diving. Fifth ed. Edinburgh: Saunders. pp. 211–226.

[26] Soto NA, Johnson MP, Madsen PT, Diaz F, Dominguez I, et al (2008) Cheetahs of the deep sea: deep foraging sprints in short-finned pilot whales off Tenerife (Canary Islands). Journal of Animal Ecology 77: 936–947. 18444999 10.1111/j.1365-2656.2008.01393.x

[27] Hochscheid S, Bentivegna F, Hamza A, Hays GC (2010) When surfacers do not dive: multiple significance of extended surface times in marine turtles. Journal of Experimental Biology 213: 1328–1337. 20348345 10.1242/jeb.037184

[28] Goldbogen JA, Calambokidis J, Croll DA, Harvey JT, Newton KM, et al (2008) Foraging behavior of humpback whales: kinematic and respiratory patterns suggest a high cost for a lunge. Journal of Experimental Biology 211: 3712–3719. 19011211 10.1242/jeb.023366

[29] Wilson RP, McMahon CR, Quintana F, Frere E, Scolaro A, et al (2011) N-dimensional animal energetic niches clarify behavioural options in a variable marine environment. Journal of Experimental Biology 214: 646–656. 21270314 10.1242/jeb.044859

[30] Williams TM, Fuiman LA, Horning M, Davis RW (2004) The cost of foraging by a marine predator, the Weddell seal Leptonychotes weddellii: pricing by the stroke. Journal of Experimental Biology 207: 973–982. 14766956 10.1242/jeb.00822

[31] Kooyman GL (1989) Diverse Divers Physiology and Behavior; Farner DS, editor. Berlin: Springer-Verlag. 200 p.

[32] Kramer DL (1988) The behavioral ecology of air breathing by aquatic mammals. Canadian Journal of Zoology 66: 89–94.

[33] Sparling CE, Fedak MA, Thompson D (2007) Eat now, pay later? Evidence of deferred food-processing costs in diving seals. Biology Letters 3: 94–98. 17443975 10.1098/rsbl.2006.0566 PMC2373816

[34] Noren DP, Williams TM, Berry P, Butler E (1999) Thermoregulation during swimming and diving in bottlenose dolphins (*Tursiops truncatus*). Journal of Comparative Physiology B 169: 93–99. 10.1007/s003600050198 10227183

[35] Williams TM, Noren D, Berry P, Estes JA, Allison C, et al (1999) The diving physiology of bottlenose dolphins (*Tursiops truncatus*). III. Thermoregulation at depth. Journal of Experimental Biology 202: 2763–2769. 10504312 10.1242/jeb.202.20.2763

